# Supplementary material for: Data Sharing in Southeast Asia During the First Wave of the COVID-19 Pandemic
Source: Front Public Health. 2021 Jun 16;9:662842. doi: 10.3389/fpubh.2021.662842 (PMC8242246; doi:10.3389/fpubh.2021.662842)
Supplement: Supplementary file 1 [file Table_1.docx]

**Supplementary Table 1. Availability of government dashboards and trackers for COVID-19, frequency of updates, and raw data sharing formats used in countries in Southeast Asia as of April 20, 2020**

| Country | Availability of government tracker/ dashboard | Frequency of update | Raw data sharing formats ever used | | | |
| --- | --- | --- | --- | --- | --- | --- |
|  |  |  | HTML | CSV | PDF | Other |
| Brunei | Available^1^ | Unclear | Yes | No | Yes | None |
| Cambodia | Available^2^ | Daily | No | No | No | None |
| Indonesia | Available^3-5^ | Daily | No | No | No | None |
| Lao PDR | Available^6^ | Unclear | Yes^7^ | No | No | None |
| Malaysia | Available^8^ | Daily | Yes^9^ | No | No | None |
| Philippines | Available^10^ | Daily | Yes^10^ | No | Yes^11^ | Google Sheets^12^ |
| Singapore | Available^13^ | Daily | Yes^14^ | No | Yes^14^ | None |
| Thailand | Available^15^ | Daily | No | Yes^16^ | No | None |
| Timor-  Leste | Not available | Unclear | No | No | No | None |
| Vietnam | Available^17^ | Daily | Yes^17^ | No | No | None |

**Supplementary References**

1. Ministry of Health (Brunei). Welcome to HealthInfo [Internet]. 2020 [cited 2020 Jun 17]. Available from: https://www.healthinfo.gov.bn/smartguide/user/login?callback=https%3A%2F%2Fwww.healthinfo.gov.bn%2F%23%2Fhome.

2. Ministry of Health (Cambodia). ប្រព័ន្ធតាមដានករណីឆ្លងកូវីដ១៩ [Internet]. 2020 [cited 2020 Jun 17]. Available from: https://covid19-map.cdcmoh.gov.kh/?fbclid=IwAR29DjPwYCLbkqtreXbXnRgsAVjOqEAA0HnX96NQnLLdeT4XKep_PDwH-3U.

3. Ministry of Health (Indonesia). Home » Info Infeksi Emerging Kementerian Kesehatan RI [Internet]. Info Infeksi Emerging Kementerian Kesehatan RI. [cited 2020 Jun 17]. Available from: https://covid19.kemkes.go.id/.

4. Gugus Tugas Percepatan Penanganan COVID-19. Home Task Force for the Acceleration of Handling COVID-19 [Internet]. covid19.go.id. 2020 [cited 2020 Jun 17]. Available from: https://covid19.go.id/.

5. Ariansyah A. Badan Nasional Penanggulangan Bencana [Internet]. BNPB. 2020 [cited 2020 Jun 17]. Available from: https://bnpb.go.id.

6. Ministry of Health (Laos). ຄະນະສະເພາະກິດ COVID-19 [Internet]. [cited 2020 Jun 17]. Available from: https://www.covid19.gov.la/index.php.

7. Ministry of Health (Laos). ກໍລະນີຕິດເຊື້ອ [Internet]. 2020 [cited 2020 Jun 17]. Available from: https://www.covid19.gov.la/index.php?r=site%2Fdetail&id=329.

8. Evozi. COVID-19 | Malaysia Outbreak Monitor | Live Updates [Internet]. COVID-19 | Malaysia Outbreak Monitor | Live Updates. 2020 [cited 2020 Jun 17]. Available from: https://www.outbreak.my.

9. Evozi. Malaysia - Statistics | COVID-19 | Malaysia Outbreak Monitor | Live Updates [Internet]. Malaysia - Statistics | COVID-19 | Malaysia Outbreak Monitor | Live Updates. 2020 [cited 2020 Jun 17]. Available from: https://www.outbreak.my/stats.

10. Department of Health (Philippines). COVID-19 Tracker Philippines [Internet]. 2020 [cited 2020 Jun 17]. Available from: https://ncovtracker.doh.gov.ph/.

11. Department of Health (Philippines). Updates of Novel Coronavirus Disease (COVID-19) [Internet]. [cited 2020 Mar 19]. Available from: https://www.doh.gov.ph/2019-nCov.

12. Department of Health (Philippines). DOH COVID-19 DataDrop - Google Drive [Internet]. 2020 [cited 2020 Jun 10]. Available from: https://drive.google.com/drive/folders/10VkiUA8x7TS2jkibhSZK1gmWxFM-EoZP.

13. Ministry of Health (Singapore). COVID-19: Cases in Singapore [Internet]. 2020 [cited 2020 Jun 17]. Available from: http://www.gov.sg/article/covid-19-cases-in-singapore.

14. Ministry of Health (Singapore). MOH | News Highlights [Internet]. 2020 [cited 2020 Jun 17]. Available from: https://www.moh.gov.sg/news-highlights/details/797-more-cases-discharged-151-new-cases-of-covid-19-infection-confirmed.

15. Digital Government Development Agency. ระดับความเสี่ยงและคำแนะนำในการปฏิบัติตน COVID19 (Update ตามประกาศ กรมฯ วันที่ 7/4/2020) - Open Government Data of Thailand [Internet]. 2020 [cited 2020 Jun 17]. Available from: https://data.go.th/en/dataset/covid19.

16. Digital Government Development Agency (Thailand). รายงาน COVID-19 ประจำวัน - Open Government Data of Thailand [Internet]. 2020 [cited 2020 Jun 17]. Available from: https://data.go.th/en/dataset/covid-19-daily.

17. Ministry of Health (Vietnam). TRANG TIN VỀ DỊCH BỆNH VIÊM ĐƯỜNG HÔ HẤP CẤP COVID-19 - Bộ Y tế - Trang tin về dịch bệnh viêm đường hô hấp cấp COVID-19 [Internet]. 2020 [cited 2020 Jun 17]. Available from: https://ncov.moh.gov.vn/.
