# Supplementary material for: Data Sharing in Southeast Asia During the First Wave of the COVID-19 Pandemic
Source: Front Public Health. 2021 Jun 16;9:662842. doi: 10.3389/fpubh.2021.662842 (PMC8242246; doi:10.3389/fpubh.2021.662842)
Supplement: Supplementary file 2 [file Table_2.docx]

**Supplementary Table 2. Variables included in the study and the definitions of ‘change in reporting’**

| **Variable** | **Definition and possible responses** | **Change in reporting** |
| --- | --- | --- |
| Date of reporting | Date at which first case of COVID-19 is reported or date at which there is change of reporting in any of the variables | Not applicable |
| Cases | Number of cases as of the ‘Date of reporting’ | Not applicable |
| Data aggregation | Type of data aggregation used. “Individual” denotes that information is reported for each individual case, “Aggregated” if otherwise. | Country no longer reports individual-level data or vice versa |
| *Demographic* |  |  |
| Age | Reporting of age of COVID-19 cases. “Exact” if age in years is reported for each individual case. “Age bracket” if the country reports age ranges. “None” if not reported. | Country’s level of reporting shifts between “Exact”, “Age bracket”, or “None” |
| Sex | Reporting of sex of COVID-19 cases. “With” if the country reports the sex of their individual confirmed cases. “Without” if not reported. | Country no longer reports data on sex after reporting it previously, or vice versa. |
| Occupation | Reporting of occupation of COVID-19 cases. “With” if the country reports the occupation of individual confirmed cases. “Without” if not reported. | Country no longer reports occupation data after reporting it previously or vice versa |
| *Geographic* |  |  |
| Domicile | Reporting of domicile of COVID-19 cases. “Precise location” if street-level addresses or exact location information are available. “City” if city addresses are available. “Province” if province/state-level addresses are available. “Country” if only country-level addresses are available. “None” if not reported. | Country changes the level of precision at which it reports its domicile data (e.g., precise location to province) |
| Travel history location | Reporting of international travel history of COVID-19 cases. “Precise location” if street-level addresses or exact location information are available. “City” if city addresses are available. “Province” if province/state-level addresses are available. “Country” if only country-level addresses are available. “None” if not reported. | Country changes the level of precision at which it reports its domicile data (e.g., precise location to province) |
| *Temporal* |  |  |
| Date of travel | Reporting of dates of international travel of COVID-19 cases. “Day” if the country reports the exact date of travel. “Month” if the country only reports the month of travel. “None” if it is not reported. | Country’s level of reporting shifts between “Day, “Month”, and “None” |
| Date of symptom onset | Reporting of date of symptom onset of COVID-19 cases. “Day” if the country reports the exact date of travel. “Month” if the country only reports the month of travel. “None” if it is not reported. | Country’s level of reporting shifts between “Day, “Month”, and “None” |
| Date of confirmation | Reporting of date of laboratory confirmation of COVID-19 cases. “Day” if the country reports the exact date of confirmation. “Month” if the country only reports the month of confirmation. “None” if it is not reported. | Country’s level of reporting shifts between “Day, “Month”, and “None” |
| Date of hospital admission | Reporting of date of hospital admission of COVID-19 cases. “Day” if the country reports the exact date of hospital admission. “Month” if the country only reports the month of hospital admission. “None” if it is not reported. | Country’s level of reporting shifts between “Day, “Month”, and “None” |
| Date of discharge, recovery, or death | Reporting of date of hospital discharge or recovery or death of COVID-19 cases. “Day” if the country reports the exact date of hospital discharge or death. “Month” if the country only reports the month of hospital discharge or death. “None” if it is not reported. | Country’s level of reporting shifts between “Day, “Month”, and “None” |
| *Other epidemiological variables* |  |  |
| Travel history | Reporting of international travel history of COVID-19 cases. “With” if the country reports the travel history of individual confirmed cases. “Without” if not reported. | Country no longer reports travel history data after reporting it previously or vice versa |
| Symptoms | Reporting of any symptoms of COVID-19 cases. “With” if the country reports the symptoms of individual confirmed cases. “Without” if not reported. | Country no longer reports symptom data after reporting it previously or vice versa |
| Comorbidities | Reporting of any chronic disease or comorbidity of COVID-19 cases. “With” if the country reports the occupation of individual confirmed cases. “Without” if not reported. | Country no longer reports comorbidities after reporting it previously or vice versa |
| Cluster | Reporting of disease clusters or known contacts of COVID-19 cases. “With” if the country reports the occupation of individual confirmed cases. “Without” if not reported. | Country no longer reports cluster data after reporting it previously or vice versa |
| Outcome | Reporting of hospital discharge or recovery or death of COVID-19 cases. “With” if the country reports the occupation of individual confirmed cases. “Without” if not reported. | Country no longer reports outcome data after reporting it previously or vice versa |
